# Evaluation of an Emergency Department-based Palliative Care Extender Program on Hospital and Patient Outcomes

**DOI:** 10.5811/westjem.56015

**Published:** 2023-05-05

**Authors:** Kalpana Narayan Shankar, Julianne Dugas, Sorraya Jaiprasert, Lauren Nentwich, Lisa Caruso, Alexandra Dobie, Deborah Whalen, Perla Macip-Rodriguez, Sandhya Rao, Suzanne E. Mitchell

**Affiliations:** * Brigham and Women’s Hospital, Department of Emergency Medicine, Boston, Massachusetts; † Boston University School of Medicine, Boston, Massachusetts; ‡ Boston Medical Center, Department of Emergency Medicine, Boston, Massachusetts; § Boston Medical Center, Department of Geriatrics, Boston, Massachusetts; || Boston Medical Center, Department of Internal Medicine, Boston, Massachusetts; # Boston Medical Center, Department of Family Medicine, Boston, Massachusetts

## Abstract

**Background:**

Boston Medical Center (BMC), a safety-net hospital, treated a substantial portion of the Boston cohort that was sick with COVID-19. Unfortunately, these patients experienced high rates of morbidity and mortality given the significant health disparities that many of BMC’s patients face. Boston Medical Center launched a palliative care extender program to help address the needs of critically ill ED patients under crisis conditions. In this program evaluation our goal was to assess outcomes between those who received palliative care in the emergency department (ED) vs those who received palliative care as an inpatient or were admitted to an intensive care unit (ICU).

**Methods:**

We used a matched retrospective cohort study design to assess the difference in outcomes between the two groups.

**Results:**

A total of 82 patients received palliative care services in the ED, and 317 patients received palliative care services as an inpatient. After controlling for demographics, patients who received palliative care services in the ED were less likely to have a change in level of care (P<0.001) or be admitted to an ICU (P<0.001). Cases had an average length of stay of 5.2 days compared to controls who stayed 9.9 days (P<0.001).

**Conclusion:**

Within a busy ED environment, initiating palliative care discussions by ED staff can be challenging. This study demonstrates that consulting palliative care specialists early in the course of the patient’s ED stay can benefit patients and families and improve resource utilization.

## INTRODUCTION

Massachusetts had the third highest prevalence of coronavirus disease 2019 (COVID-19) after New York and New Jersey with a total of 72,025 cases and 4,420 deaths, as of May 6, 2020.^1^ Boston and the surrounding municipalities were disproportionately affected with 21.2% of the state’s COVID-19 cases diagnosed.^1^ As New England’s largest safety-net hospital, serving primarily low-income individuals and those who are defined as racial and ethnic minorities, Boston Medical Center (BMC) saw a surge of COVID-19 patients. This population has historically experienced significant disparities in health and outcomes, including elevated rates of morbidity and mortality from chronic disease, which added to the increased risk of poor outcomes from COVID-19.^2^

In response to the rapidly rising number of patients whose health was seriously impacted by COVID-19, in April 2020 BMC established a palliative care working group to address the high mortality from the disease. From a series of informal interviews with emergency department (ED) staff, the working group identified the ED’s concern about adequately addressing the needs of critically ill patients and their family members under crisis conditions, as well as the unmet needs of patients and families for counseling, education, and advance care planning. Furthermore, with the discussion of crisis standards of care looming across the United States, the BMC ED staff was not equipped to initiate and handle such conversations in a patient-centered manner while caring for the multitude of sick patients in an already crowded ED. Therefore, BMC established a working group to create an all-volunteer Palliative Care Extender Team (PCXT), to initiate palliative care consults and advance-care planning discussions in the ED for any patient who was identified as benefitting from this additional support.

For this program evaluation we used a matched, retrospective cohort study design to assess the difference in outcomes between those who received palliative care in the ED vs those who received palliative care as an inpatient or were admitted to the intensive care unit (ICU) without a palliative care consult. Our primary outcome was length of stay (LOS). Our secondary outcomes followed intensity of services (using LOS in the ICU vs floor vs discharge disposition) and change in level of care. We hypothesized that patients who had palliative care services initiated in the ED would have a shorter overall LOS and a decrease in intensity of services compared to their matched controls.

## METHODS

### Palliative Care Extender Team Program

The PCXT was an all-volunteer group of 48 health professionals consisting of a dyad team of a physician or midlevel practitioner (advanced practice nurse or physician assistant) and a licensed clinical social worker (LICSW) or licensed clinical mental health counselor (LMHC) implemented in response to the COVID-19 pandemic surge from April 10–June 30, 2020. The dyad teams staffed two ED shifts daily from 8 am–10 pm, providing serious illness conversations and palliative care support for patients presenting to the ED with COVID-19 symptoms. Attending emergency physicians could initiate the palliative care consult based on clinical discretion for patients who they felt were moderately to severely ill from COVID-19 or if they felt the patient needed more urgent advanced care planning. No strict inclusion or exclusion criteria was implemented as this began as a program and not a research study. The dyad teams implemented palliative care assessments and serious illness conversations with patients and families and completed a documentation and billing template through the electronic health record (EHR) (Epic Systems Corporation, Verona, WI). We used the Strengthening the Reporting of Observational Studies in Epidemiology (*STROBE*) statement guidelines to ensure the reporting of this observational study.^3^

**Population Health Research Capsule**

What do we already know about this issue?

*Palliative care improves outcomes when initiated early. It improves patient and family satisfaction with symptoms management and improves quality of life*.

What was the research question?

*We sought to assess the impact on patient outcomes of initiating palliative care services in the ED*.

What was the major finding of the study?

*Patients in the intervention had shorter length of stay (5.2 vs 9.9 days), were less likely to have a change in level of care (36% vs 68%) or be admitted to an ICU (20% vs 63%, all P<0.0001)*.

How does this improve population health?

*Consulting palliative care specialists during an ED stay can improve resource utilization with shorter hospital stays, fewer changes in level of care, and fewer ICU stays*.

### Palliative Car Extender Team Professionals and Training

The clinician volunteers came from internal medicine, family medicine, and pediatrics, and the psychosocial support members came from social work, behavioral health, child health, patient advocacy, and chaplaincy departments, all of whom normally practice in the ambulatory care setting. The volunteers received a one-hour virtual orientation and training around palliative care and addressing primary palliative care needs. These included communication skills training and pocket-guide resources based on evidence-based models of serious illness communication,^4^,^5^ as well as a walk-through of the PCXT logistics and education concerning palliative-care symptom assessment and safety protocols in the ED. Concurrently, the working group created training materials and conducted rapid cycle testing of an ED-embedded workflow to guide them. The volunteer dyads were also oriented to key documentation elements to be included in the patients’ charts regarding any discussions about advance directives and goals of care. A documentation and billing template through the EHR was developed and used to document and track encounters. The teams staffed two ED shifts daily from 8 am–4 pm and 2 pm–10 pm.

### Intervention Workflow

The PCXT implementation team informed ED clinicians of the new PCXT services through departmental meetings and emails. The teams also provided in-service education on how to request a PCXT consult. The PCXT was available for consults of any COVID-19-positive patient seeking emergency care. Consult requests and their associated orders in the EHR were placed by emergency physicians. The PCXT conducted in-person and telephonic discussions with patients, emergency clinicians, healthcare proxies, and family members about goals of medical care in the setting of a pending or confirmed COVID-19 diagnosis. The PCXT also provided guidance on symptom management to maximize patient comfort and directly supported patients and family members through phone updates. Each consult took approximately one hour to complete. Consult teams would continue to follow these patients as inpatients.

### Evaluation Design

We used a matched, retrospective cohort study design to compare outcomes between those who received palliative care in the ED via the PCXTs to those who did not receive the PCXT intervention in the ED. Our primary outcome was hospital LOS (outcome); our secondary outcomes included change in level of care and intensity of services (ICU vs floor vs discharge disposition). We matched patients on age and gender. This program evaluation was approved by the BMC Institutional Review Board, protocol H-40627.

### Data Collection

We collected patient logs for the cases from the PCXT database where services were delivered in the ED. This data was already collected through an ongoing quality improvement initiative to monitor those who obtained such services. This data was then verified through BMC’s Clinical Data Warehouse (CDW). Due to the overall volume of patients in the hospital during the initial pandemic, we were unable to match an adequate sampling of control cases; thus, the controls we used were COVID-19 patients admitted to the ICU without an ED palliative care consult or those who received their palliative care consult only as an inpatient. The CDW provided all relevant patient characteristics and outcomes for both cases and controls.

### Outcomes

We defined cases as COVID-19 patients who received palliative care services in the ED due to the palliative care extender program. Controls were those COVID-19 patients who either received palliative care as an inpatient or were admitted to the ICU and received a palliative care consult only after arrival to the ICU from March 1–June 30, 2020. All included patients were at least 22 years old as this is the general cutoff age for patients seen in the adult ED. Due to staffing constraints, the palliative care extender program was not available in the pediatric ED and, thus, we excluded pediatric patients from the controls.

We obtained patient demographics including age, race, ethnicity, gender, and primary insurance through the CDW for the controls and through a combination of the CDW and ED registration for the cases. Information obtained through ED registration happened via phone conversation into patient rooms to minimize exposure of registration staff to COVID-19 patients. We also assessed hospital visit-specific data, including overall LOS, ICU LOS, and discharge disposition (to be used as a proxy for intensity of services). Code status, defined as either full code, do not resuscitate (DNR), do not intubate (DNI) or comfort measures only (CMO), was obtained via review of EHR notes. A patient’s status was ultimately based on their most recent code status.

Primary insurance was defined as either commercial, Medicaid, Medicare, managed care, or other. Discharge disposition was defined as deceased, discharged home (including with home healthcare services), discharged to hospice/other facility, or other.

### Analysis

We calculated basic descriptive statistics to summarize the outcome measures, as well as demographic information for all participants and separately by intervention group. Continuous characteristics were summarized by using means and SD or using medians and interquartile ranges (IQR) if non-normally distributed. We summarized dichotomous and categorical variables using frequencies and percentages. Patient-level statistics were calculated per patient, and visit-level statistics were calculated per visit. We used chi-squared and Wilcoxon ranked-sum tests to compare primary and secondary outcomes between cases and controls.

We then used simple and multivariable linear mixed models with random effects to compare overall LOS, ICU LOS, changes in level of care and discharge disposition between cases and controls while accounting for multiple visits per some patients. Models were produced first without covariates and then separately with the addition of patient age, race/ethnicity, and gender.

## RESULTS

A total of 82 patients who received palliative care services in the ED were compared to the 317 patients who received palliative care services as an inpatient. No patients in either group were discharged home with home hospice services as there were no hospice services for COVID-19 patients this early in the pandemic. Patients who received palliative care services in the ED were predominantly male (43, 52.4%) and non-Hispanic Black (47, 57.3%), with an average age of 76.3 years (SD 13.7). The majority (51/82, 62.2%) had only one admission during the study period. These demographics were consistent with the national trend during this period, whereby the US Centers for Disease Control and Prevention noted COVID-19 to disproportionately affect males and non-Hispanic Blacks.^6^

Managed care and Medicare were the most frequent primary insurance types (46.3% and 41%, respectively) among this group, and they predominantly carried a full code (24) or DNI/DNR (27) status. Patients who received palliative care services as an inpatient were also predominantly male (204, 64.4%), had an average age of 59.3 years (SD 17.3), were non-Hispanic Black (137, 43.2%), and had one admission in the study period (252, 79.5%). The majority also carried a full code status (204, 64.8%) or DNR (50, 15.9%) or both a DNI/DNR status (38, 12.1%). The control group was significantly younger (*P*<0.001) and had more admissions (*P*<0.001). In addition, patient type was significantly associated with primary insurance type (*P*<0.001) and code status (*P*<0.001) (Table 1).

Patients who received palliative care services in the ED were less likely to have a change in level of care (*P*<0.001) or be admitted to an intensive care unit (*P*<0.001) compared to controls. When adjusting for age, race/ethnicity, insurance and gender, cases had an average LOS of 5.2 days compared to controls who stayed 9.9 days (*P*<0.001), amounting to a difference of 4.7 days. With regard to intensity of services as measured by ICU visits, after controlling for age, race/ethnicity, insurance and gender, cases averaged fewer total number of ICU admissions (29) as compared to controls (274) (*P*<0.001). However, there was no difference in ICU LOS with a median number of five days in both groups (*P*=0.83). Discharge disposition did not differ significantly between cases and controls (*P* = 0.52). Code status was not included in the multivariable models because we could not be sure whether the code status extracted was the status at the time of their initial ED presentation or if this was a change when they were admitted. (See Table 2).

## DISCUSSION

This study demonstrated that patients for whom palliative care services were initiated in the ED were less likely to have a change in level of disposition while admitted when compared to controls, had significantly shorter LOS and fewer admissions to an ICU. This is in line with prior studies demonstrating improved outcomes with early initiation of palliative care, specifically improved satisfaction for patients and families and symptom management.^7^–^10^ Specifically, Grudzen et al and Meier et al noted improvements in quality of life for those who received early palliative care services.^7^,^8^ Additionally, Wu et al noted shortened hospital stays of less by 3.6 days with early initiation of such services,^9^ and Temel et al found improved quality of life.^10^

In this study we also noted that both groups had similar rates of commercial insurance; however, the cases were comprised primarily of Medicare patients, whereas the controls carried similar frequencies of Medicare and Medicaid. There was also a higher rate of patients with DNR/DNI or CMO status among the PCXT patients as compared to the controls. Based on this, we can surmise that older patients who were likely sicker were engaging with the PCXT in the ED, as compared to the controls. While this is not surprising given the known increased severity of sickness in the older population,^11^ this does underscore the importance of initiating palliative care discussions among sick patients in the ED with trained clinicians.

During the height of the COVID-19 pandemic, BMC received most of the symptomatic cases in the city of Boston. Given the significant stress the ED faced, a palliative care extender program was initiated to remove some of the burden of initiating discussions on goals of care and symptom management for patients who would be admitted to the hospital. To our knowledge, the BMC Palliative Care Extender Program is one of only a few novel responses implemented by a safety-net healthcare system to meet the immediate demands for palliative care in the ED during the early days and weeks of the COVID-19 pandemic.

A 2014 meta-analysis estimated that palliative care consultation reduced hospital inpatient costs by 10–30%.^12^ Clearly, palliative care services provide an invaluable benefit to hospitals, patients, and families that would otherwise be difficult to achieve with ED staff alone, given the time and resource constraints of initiating and engaging in these vital, high-stakes discussions. Furthermore, physicians who can palliative care should be able to connect with patients in an unbiased manner to enter into shared decision-making processes on goals of care and appointment of a healthcare proxy. Too often, ED staffing constraints limit the reach of palliative care services to inpatient or ambulatory settings.

This program was unique in that there was a physician or a mid-level clinician with a licensed mental health clinician who received palliative care training and resources to initiate advance care planning in a setting where this discussion is frequently initiated by those with limited palliative care training. This dyad was able to provide support and guidance to emergency clinicians on symptom management in a way that allowed the ED staff to efficiently run the department without taking away from the sensitivity of advance care planning. Lastly, because these dyads work throughout a hospital system, they can provide continuous support throughout an entire hospitalization to patients and family members through phone calls and meetings regarding a patient’s change in status—something emergency clinicians are unable to do.

To date, few palliative care service programs operate in the ED setting. Those EDs that have implemented such services noted an increase in educational awareness and confidence of palliative care delivery, but time constraints and implementation logistics were their biggest limitation.^13^,^14^ Despite the ongoing education of emergency physicians and movements through national emergency medicine societies to increase palliative care education, emergency physicians still often find it difficult to engage in these discussions, accurately predict a disease process, or foreshadow outcomes of an invasive procedure.^15^ With the many competing interests (dealing with a variety of acutely ill patients, unpredictable ED volumes, lack of dedicated time and training), emergency clinicians face many challenges regarding initiating and completing advance care planning discussions in a meaningful way that does not feel rushed by the patient. The extender model developed in this program was able to address these barriers and provide patient-centered care in a clinically appropriate manner.

## LIMITATIONS

As we were limited by the true number of patients who received this service, this was a small sample size; larger numbers would have provided more power. Second, we were unable to match an adequate sampling of controls to cases due to overall low hospital volumes during this time; thus, over half of our cases had no control. Because of this, we were unable to match on other important factors such as level of admission, comorbidities, or illness severity, which are reflected in the differences in some of the baseline characteristics between groups. However, based on the sample size available to us, we controlled for as many variables as possible in the multivariate analysis. Third, we did not control for comorbidity, which limited our ability to assess whether underlying sickness contributed to differences in their outcomes.

While there was no difference in death and discharge disposition to suggest that patient severity could be similar for both groups, the age difference between the groups could also indicate the opposite. As previously mentioned, hospital volumes limited our ability to obtain an adequate, comparative sample. Fourth, there may have been variation in volunteer comfort in providing elements of palliative care, especially for those who do not exclusively practice palliative care or typically deal with serious illness conversations, which could have led to a difference in outcomes. However, if this was an overriding theme, the outcomes would likely have been biased toward the null. Lastly, this data was taken from an urban, academic hospital using volunteers; thus, results and true costs of such an intervention may not be generalizable for community settings where palliative care services are not always available.

## CONCLUSION

The ED may not seem the ideal place to initiate sensitive discussions regarding patients’ and families’ goals and values regarding their healthcare. However, it may also be the most meaningful place to do so, especially given the isolation and prognostic uncertainty that patients and families faced surrounding COVID-19. This study demonstrates that consulting palliative care specialists early in the patient’s course of their ED stay can improve resource utilization with shorter hospitalization stays, fewer changes in level of care, and fewer ICU stays. It also offloaded this delicate conversation from members of the ED care team who are often multitasking other patients and procedures to an independent team who had time to focus on a conversation about serious illness. Future studies should follow usage and further assess the barriers to replicating such models or consulting palliative care services in the ED.

## Figures and Tables

**Table 1:** Baseline characteristics of patients receiving palliative services during COVID-19.

	Patients who received palliative services in the ED (n=82)	Patients who had palliative services initiated as an Inpatient (n=317)	P-value
Gender, n (%)			0.05 (chi-square)
Female	39 (47.6)	113 (35.6)	
Male	43 (52.4)	204 (64.4)	
Age (years), mean (SD)	76.3 (13.7)	59.3 (17.3)	<0.001 (t-test)
Race/ethnicity, n (%)			0.02 (chi-square)
Black, non-Hispanic	47 (57.3)	137 (43.2)	
White, non-Hispanic	17 (20.7)	51 (16.1)	
Hispanic	14 (17.1)	102 (32.2)	
Other	3 (3.7)	8 (2.5)	
Missing	1 (1.2)	19 (6.0)	
Admissions per patient, n (%)			<0.001 (Fisher exact)
1	51 (62.2)	252 (79.5)	
2	14 (17.1)	41 (12.9)	
3	11 (13.4)	9 (2.8)	
4	2 (2.4)	11 (3.5)	
5	2 (2.4)	0 (0.0)	
6	1 (1.2)	1 (0.3)	
7	0 (0.0)	0 (0.0)	
8	0 (0.0)	1 (0.3)	
9	1 (1.2)	1 (0.3)	
10	0 (0.0)	1 (0.3)	
Primary insurance, n (%)			<0.001 (chi-square)
Commercial	0 (0.0)	21 (6.6)	
Managed care	38 (46.3)	143 (45.1)	
Medicaid	8 (9.8)	62 (19.6)	
Medicare	34 (41.5)	64 (20.2)	
Other	2 (2.4)	26 (8.2)	
Missing	0 (0.0)	1 (0.3)	
Code status			<0.001 (chi-square)
Full code, n (%)	29 (36.7)	209 (66.4)	
DNI, n (%)	3 (3.8)	3 (1.0)	
DNR, n (%)	4 (5.1)	53 (16.8)	
Both DNI/DNR, n (%)	28 (35.4)	39 (12.4)	
CMO, n (%)	15 (19.0)	11 (3.5)	

*ED*, emergency department; *nH*, non-Hispanic; *DNI*, do not intubate; *DNR*, do not resuscitate; *CMO*, comfort measures only.

**Table 2:** Adjusted outcomes.

	Patients who received palliative services in the ED (n=82)	Patients who had palliative services initiated as an inpatient (n=317)	Test	P-value
LOS (days), median (IQR)	5.2 (7.2)	9.9 (13.5)	Wilcoxon ranked-sum test	<0.001
Visits with ICU stays, n (%)	29 (20.1)	274 (63.4)	chi-squared	<0.001
ICU LOS (days), median (IQR)	5.0 (8.0)	5.0 (11.0)	Wilcoxon ranked-sum test	0.83
Change in level of care (either upgraded or downgraded while inpatient), n (%)	52 (36.1)	294 (68.1)	chi-squared	<0.001
Discharge disposition, n (%)			chi-squared	0.52
Home	62 (43.1)	199 (46.1)		
Other medical facility	16 (11.1)	80 (18.5)		
Deceased	23 (16.0)	83 (19.2)		
Other	3 (2.1)	8 (1.9)		

*ED*, emergency dexpartment; *LOS*, length of stay; *IQR*, interquartile range; *ICU*, intensive care unit.
